# Perioperative complications of abdominal surgery in smokers

**DOI:** 10.1007/s00540-020-02815-6

**Published:** 2020-06-23

**Authors:** Yoshikazu Inoue, Takahiko Katoh, Shota Masuda, Xi Lu, Tadashi Koga, Tomohiro Sadohara, Michiaki Sadanaga, Eiji Tanaka

**Affiliations:** 1 Department of Anesthesiology, Japanese Red Cross Kumamoto Hospital, 2-1-1 Nagamineminami, Higashi-ku, Kumamoto, 861-8528 Japan; 2 Department of Public Health, Faculty of Life Sciences, Kumamoto University, 1-1-1 Honjou, Chuo-ku, Kumamoto, 860-8556 Japan; 3 Department of Surgery, Japanese Red Cross Kumamoto Hospital, 2-1-1 Nagamineminami, Higashi-ku, Kumamoto, 861-8528 Japan

**Keywords:** Smoking cessation, Postoperative complications, Abdominal surgery

## Abstract

**Purpose:**

This study examined the association between smoking and perioperative complications of laparoscopic abdominal surgery and whether these complications were reduced with ≥ 4 weeks of preoperative smoking cessation.

**Methods:**

A total of 555 patients who underwent gastric and colorectal cancer surgeries under general anesthesia were divided into the following groups retrospectively: 290 individuals without smoking history (NS group), 144 previous smokers (stopped smoking more than 8 weeks before surgery, PS group), and 121 current smokers (CS group) divided to two groups according to preoperative smoking cessation for < 4 (CS1, *n* = 76) and 4–8 weeks (CS2, *n* = 45).

**Results:**

When compared with the NS group, postoperative hospitalization duration was significantly longer in the CS1 group (*p* < 0.01), whereas differences between the CS2 or PS groups and NS group were not significant. The total number of postoperative complications was higher in all groups of smoking than in NS group, independent on preoperative smoking cessation; however, suture failure was significantly more frequent only in CS1 group. Although pack-years did not significantly affect complication rates in smokers, duration of smoking cessation time in PS group was a negative predictor of postoperative complications.

**Conclusion:**

Providing more than 4 weeks of smoking cessation before gastrointestinal surgery can reduce the duration of hospitalization and rate of suture failure.

## Introduction

Previous research has identified smoking as an obvious cause of malignant diseases, such as laryngeal, esophageal, and lung cancer; cardiovascular disorders; and diabetes [1–3]. Apart from primary disease, cancer patients who smoke also experience systemic diseases caused by smoking, and considerable effects of smoking on surgery. Previous studies have emphasized smoking as a risk factor for different kinds of postoperative complications in various types of patients and surgeries, including gastrointestinal surgery [4–8]. Interventions of preoperative smoking cessation reduced postoperative complications in different kinds of patients [7–13]. In some studies, the proportions of surgeons, anesthesiologists abroad [14], thoracic surgeons, and anesthesiologists in Japan [15] who actively provided preoperative smoking cessation guidance were approximately 58%, 30%, 26%, and 6%, respectively; hence, it is recommended to further highlight the benefits of this approach.

Although most studies showed positive effects of preoperative smoking cessation, some did not find significantly improved outcomes [16, 17]. These inconsistencies likely reflect the differences in duration of preoperative smoking cessation, variability in inclusion criteria (different patients, different types of surgery) and different endpoints (all complications, special type of complications, mortality). In this context, it is still unclear how long period of smoking cessation before gastrointestinal surgery is needed to reduce postoperative complications and reduce hospital stays. The minimum reported beneficial durations of smoking cessation before surgery were 2 weeks [8], 3 weeks [18], 4 weeks [19], or 6–8 weeks [20]. In gastric cancer surgery, at least 2 weeks of smoking cessation [8] helped reduce the incidence of postoperative complications, whereas another study [7] reported lower postoperative pulmonary problems in patients who stopped smoking ≥ 4 weeks before surgery. Meanwhile, for patients undergoing colorectal surgery, short-term cessation (2–3 weeks) failed to improve tissue and wound healing or avoid other complications [17].

Here, we hypothesized that rates of postoperative complications and duration of hospital stays decline with longer smoking cessation times before surgery. The aim of this study was to compare the postoperative complication rate and duration of postoperative hospital stays (as primary endpoints) between patients who stopped smoking for different time periods before the surgery (less than 4 weeks, 4–8 weeks, and > 8 weeks) and patients who never smoked. The secondary objective was to assess the effects of pack-years and exact smoking cessation duration on complication rates.

## Methods

In this retrospective, observational cohort study, we collected clinical data of patients with the American Society of Anesthesia physical status classification of 1/2, who had undergone laparoscopic abdominal surgery for gastric and colon cancers. The study was conducted at Kumamoto Red Cross Society, a regional hospital in Japan. The institutional ethics committee approved the study protocol (#268), and patient confidentiality was maintained throughout the study. Opt-out was used because it was an observational study in accordance with ethical guidelines for humans.

**Fig. 1:**
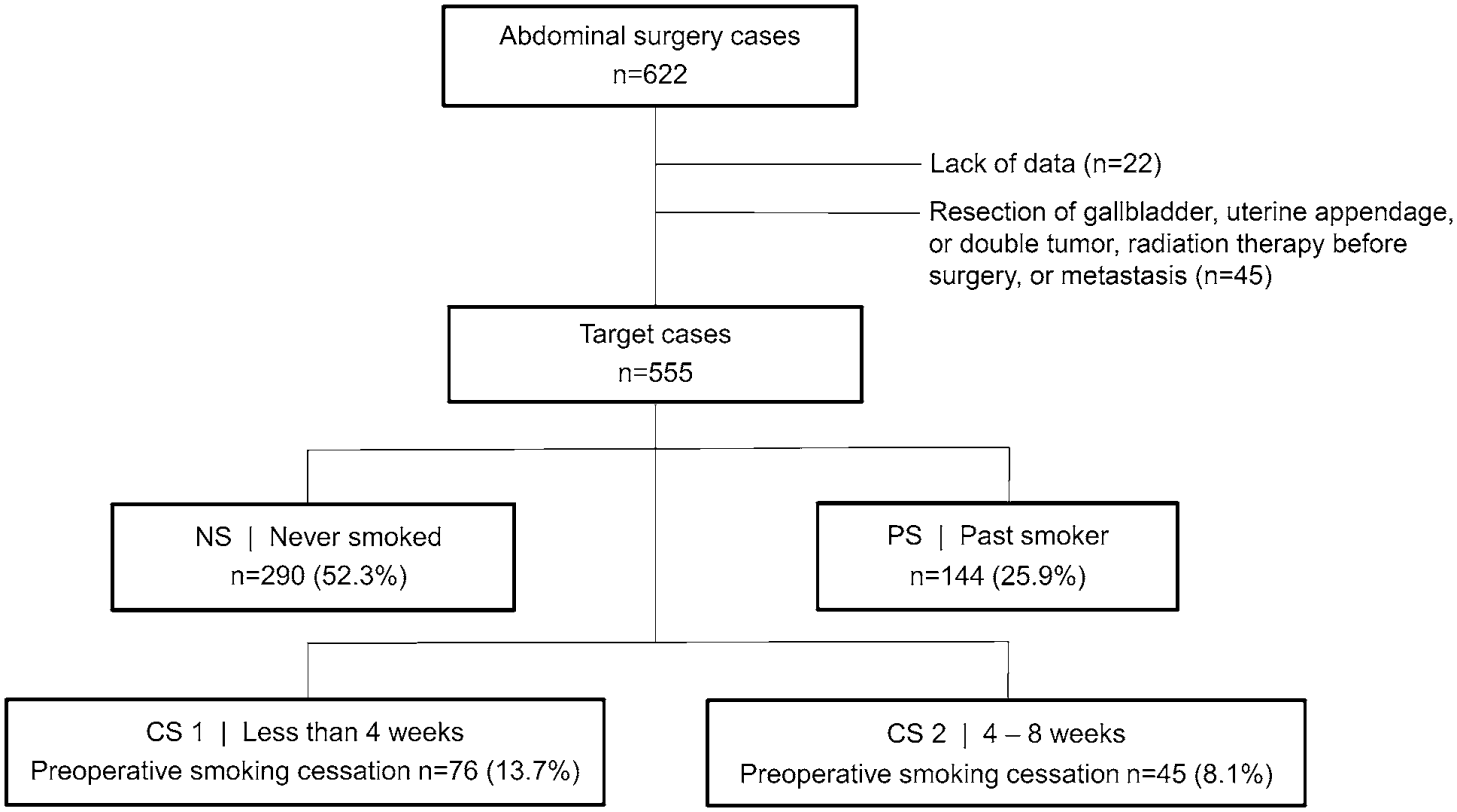
Flowchart of the study

All patients who had undergone gastric and colorectal cancer laparoscopic surgeries under general anesthesia between January 2015 and December 2019 were considered (*n* = 622); among them, 45 cases with resection of gallbladder, uterine appendage, or double tumor, radiation therapy before surgery, or metastasis, and 22 patients lacking preoperative smoking information were excluded. Finally, 555 cases were included in the study. We divided the participants into four groups according to smoking status at the time of operation: patients who stopped smoking < 4 weeks before the surgery (CS1, *n* = 76), those with smoking cessation 4–8 weeks before the surgery (CS2, *n* = 45), those with smoking cessation > 8 weeks before the surgery (PS group, *n* = 144), and those who never smoked (NS group, *n* = 290) (Fig. 1). Age, sex, body mass index, FEV1/FVC ratio, time under anesthesia, bleeding volume, incidence of postoperative complications, and postoperative hospitalization duration were compared among these groups. The primary endpoints were the incidence of postoperative complications and duration of postoperative hospital stay. Secondary endpoints were correlations between the pack-years and occurrence of complications in all smoking groups, and between smoking cessation period and complications in the PS group.

Postoperative complications were recorded in accordance to Clavien–Dindo classification of postoperative complications proposed by Dindo et al. [21]. Specifically, we recorded complications with grade II or higher, which involve complications requiring postoperative medication other than antiemetics, antipyretics, analgesics, and diuretics. Postoperative complications were divided into respiratory (pneumonia, pleural effusion, and atelectasis); digestive tract (suture failure and intestinal obstruction); circulatory (arrhythmia, thrombosis, and angina pectoris), surgical wound (infection); and other (delirium, urinary tract infections, and unknown fever) complications. The complications were recorded from the medical records and were based on the clinical, biochemical, and/or radiological manifestations. We considered postoperative complications and site, presence or absence of diabetes, and alcohol consumption as potential confounding variables.

Statistical analyses were performed using EZR version 2.4–0 (Saitama Medical Center, Jichi Medical University, Saitama, Japan [22])—a graphical user interface for R (The R Foundation for Statistical Computing, Vienna, Austria). Continuous data were expressed as means and standard deviations. Student’s *t* test was used to compare continuous variables and chi-square test to compare categorical variables. Inter-group comparisons were performed using one-way ANOVA, and Tukey’s post hoc test was used for multiple comparisons. Adjusted odds ratios were calculated using multivariate logistic regression to control for potential confounders. Statistical significance was set at *p* < 0.01.

## Results

Among 555 patients, 450 (81.1%) used inhaled anesthetics and 105 (18.9%) underwent general anesthesia with TIVA. Epidural anesthesia was not used in 26 cases (4.7%). In patients who did not use epidural anesthesia for postoperative analgesia, continuous infusion of fentanyl citrate was performed after surgery. Gastric cancer surgery was performed by nine surgeons [one surgeon performed surgeries in 155/191 (81.2%) cases]. Colorectal cancer surgery was performed by 12 surgeons [two surgeons performed surgeries in 288/364 (79.1%) cases].

The CS1 group was significantly younger than the PS and NS groups (Table 1). The proportion of men in NS group was significantly lower than in each smoking group, while CS1 group additionally showed lower percent of men as compared to CS2 and PS groups (Table 1). The average FEV1/FVC ratios were significantly lower in each smoking group compared to the NS group (*p* < 0.01; Table 1). Total preoperative complications, surgical time, anesthesia time, and bleeding volume did not differ significantly among the groups. However, incidence of postoperative complications was significantly higher in the CS1 and PS groups than in the NS group (*p* < 0.01; Table 1). Postoperative hospitalization duration was significantly longer in the CS1 group compared with NS group (*p* < 0.01; Table 1). Logistic regression analysis showed that the odds for all kinds of complications were affected by smoking (*p* < 0.01; Table 2). When considering only suture failure as complications, only the CS1 group showed significantly more complications, both before and after adjusting for age, diabetes, alcohol consumption, anesthesia time, and surgical site (Table 2).

**Table 1:** Basic demographic and clinical characteristics of included patients (*n* = 555)

Variable	Group				*P* value
	NS (*n* = 290)	CS 1 (*n* = 76)	CS 2 (*n* = 45)	PS (*n* = 144)	
Age (years)	70.4 ± 10.9	64.9 ± 11.5	65.9 ± 10.6	69.5 ± 10.9	< 0.001*^, ||^
Sex					< 0.001*^, †, ‡, §, ||^
Male	102 (35.2)	80 (76.9)	38 (84.4)	130 (90.3)	
Female	188 (64.8)	24 (23.1)	7 (15.6)	14 (9.7)	
Body mass index	23.0 ± 4.0	22.6 ± 3.1	23.4 ± 3.1	23.6 ± 3.9	0.373
Pack-years	/	36.3 ± 18.2	41.2 ± 23.3	34.3 ± 22.6	0.103
Preoperative complications	203 (70.1)	53 (69.7)	31 (68.9)	109 (75.7)	0.631
HT	106 (52.2)	14 (26.4)	13 (41.9)	47 (43.1)	< 0.01*
DM	16 (7.9)	6 (11.3)	5 (16.1)	9 (8.3)	0.405
HT + DM	33 (16.3)	14 (26.4)	4 (12.9)	21 (19.3)	0.326
COPD	0 (0)	3 (5.7)	1 (3.2)	5 (4.6)	0.014
FEV1.0 / FVC (%)	81.3 ± 6.4	77.3 ± 7.9	76.8 ± 7.9	77.4 ± 5.6	< 0.001 ^*, †, ‡^
Surgical time (min)	271 ± 83	284 ± 122	296 ± 73	285 ± 94	0.291
Anesthesia time (min)	328 ± 85	345 ± 126	349 ± 73	346 ± 97	0.230
Bleeding volume (ml)	100 ± 254	84 ± 151	135 ± 258	93 ± 217	0.803
Postoperative complications	17 (5.9)	21 (27.6)	7 (15.5)	29 (20.1)	< 0.001*^, ‡^
Suture failure	4 (1.4)	8 (10.5)	1 (2.2)	3 (2.1)	< 0.01*
Other complications	13 (4.5)	13 (17.1)	6 (13.3)	26 (18.0)	< 0.001*^, ‡^
Intestinal obstruction	4 (23.5)	1 (4.8)	4 (57.1)	6 (20.7)	
Pneumonia	2 (11.8)	2 (9.5)	0 (0)	3 (10.3)	
Arrhythmia	0 (0)	1 (4.8)	0 (0)	2 (6.9)	
Postoperative hospitalization duration (days)	9.5 ± 4.0	12.2 ± 10.9	11.6 ± 5.9	11.3 ± 7.5	< 0.01 *

Values are presented as *n* (%) or mean ± SD

*NS *patients without smoking history, *CS 1-* current smokers who stopped smoking less than 4 weeks prior to surgery, *CS 2-* current smokers who stopped smoking 4–8 weeks prior to surgery, *PS *past smokers (stopped smoking more than 8 weeks prior to surgery); Preoperative complications (obtained from the description in the medical records): *HT *hypertension, *DM *diabetes mellitus, *COPD *chronic obstructive pulmonary disease, others (e.g., bronchial asthma, chronic kidney disease, atrial fibrillation)

*FEV1.0 *forced expiratory volume in one second, *FVC *forced vital capacity

Postoperative complications were recorded as grade II or higher in Clavien–Dindo classification of postoperative complications [21]

Significant post hoc pairwise comparisons (*P* < 0.05)

*NS versus CS1, ^†^NS versus CS2, ^‡^NS versus PS, ^§^CS1 versus CS2, ^||^CS1 versus PS

**Table 2:** Odds ratios (95% confidence intervals for OR) for the presence of postoperative complications depending on the smoking status and confounding variables

Predictor variable	Complications	Groups		
		CS 1 (*n* = 76)	CS 2 (*n* = 45)	PS (*n* = 144)
Smoking status	All complications	5.54 [ 2.73–11.2]*	4.01 [1.67–9.67]*	3.89 [2.01–7.55]*
	Suture failure	8.17 [ 2.39–27.9]*	3.33 [0.59–18.7]	1.79[0.39–8.11]
Smoking status *adjusted*^#^	All complications	7.53 [3.08–18.7]*	5.95 [1.99–17.8]*	5.30 [2.28–12.3]*
	Suture failure	8.83 [1.55–50.3]*	5.78 [0.66–50.4]	2.25 [0.31–16.5]

^#^Adjusted for age, diabetes, alcohol, anesthesia time, and surgical site

*CS 1-* current smokers who stopped smoking less than 4 weeks prior to surgery, *CS 2-* current smokers who stopped smoking 4–8 weeks prior to surgery, *PS *past smokers (stopped smoking more than 8 weeks prior to surgery)

*p < 0.01

The pack-years of each smoking group are shown in Table 1. The presence of complications did not depend on pack-years in any group (*p* > 0.05 for all). In the PS group, smoking cessation period was 17.9 ± 13.3 years, and there was significant negative correlation between the presence of complications and duration of smoking cessation until surgery (*p* < 0.01) (Table 3).

**Table 3:** Odds ratios (95% Confidence Intervals for OR) for the presence of postoperative complications based on the pack-years and duration of smoking cessation

Predictor variable	Group		
	CS 1 (*n* = 76)	CS 2 (*n* = 45)	PS (*n* = 144)
Pack-years	1.00 [0.98–1.02]	0.98 [0.93–1.02]	1.01 [0.99–1.03]
Duration of smoking cessation (years)	–	–	0.93 [0.88–0.97]*

*CS 1-* current smokers who stopped smoking less than 4 weeks prior to surgery, *CS 2-* current smokers who stopped smoking 4–8 weeks prior to surgery, *PS—*past smokers (stopped smoking more than 8 weeks prior to surgery)

*p < 0.01

## Discussion

In this study we explored whether preoperative smoking cessation was able to reduce postoperative complications after laparoscopic gastrointestinal surgery. In contrast to those who stopped smoking < 4 weeks before surgery, individuals who stopped smoking more than four weeks before the surgery had comparable duration of hospitalization and number of suture failure complications as non-smokers.

Patient’s age was significantly lower in the group with < 4 weeks smoking cessation compared to PS and NS groups, suggesting that cancer occurred at significantly younger age. Smoking is a potential risk factor for gastric and colon cancers. FEV1/FVC ratio was significantly higher in the NS than in CS1, CS2, and PS groups, likely reflecting that smokers have first signs of obstructive pulmonary diseases, such as pulmonary emphysema. All smoking groups showed higher occurrence of all types of complications than NS group, which corresponds to previous studies regarding digestive surgery that showed increased frequency of respiratory complications and postoperative suture defects in smokers [23, 24] and likely adverse effects on wound healing. Indeed, smoking is an independent risk factor for postoperative suture failure [4–6] because of impaired blood flow at the anastomotic site, which occurs because of vasoconstrictive action of tobacco smoke including nicotine [25, 26]; this could explain many suture failure cases. In contrast to Jung et al. where even preoperative smoking cessation for at least 2 weeks helped to reduce postoperative complications in gastric cancer surgery [8], we observed no improvements under 4 weeks. In another study in gastric cancer patients, 4 weeks of smoking cessation were sufficient to reduce pulmonary complications [7]. However, in our study, considering all postoperative complications, all three smoking groups had more complications than the NS group, suggesting that even after longer smoking cessation some of the negative effects of smoking remain, predisposing to some of the complications. However, statistical significance was achieved only for the CS1 vs. NS group, and for the PS vs. NS group comparisons, whereas comparisons of CS2 with NS or PS were not significant. Lack of significant differences in postoperative complications between CS2 and NS groups, and CS2 and PS groups, may stem from a relatively small size of CS2 group. Considering a large standard deviation in smoking cessation period for the PS group (17.9 ± 13.3 years), some of those patients had a smoke-free period close to that in CS2 group, additionally contributing to lack of differences. Furthermore, negative correlation between the presence of complications and duration of smoking cessation before surgery in the PS group suggests that smoking cessation before surgery may be more important than pack-years in reducing postoperative complications. Although postoperative complications were significantly higher in the PS compared with NS group, it was evident that the PS group actually showed comparable number of typical postoperative complications, but a higher number of other complications (Table 1). Hence, the definition of postoperative complications should be considered when interpreting the results. In addition, although not significantly different, the higher rate of preoperative complications in the PS group may have also contributed to its increased postoperative complications.

Nevertheless, if focusing on suture failures alone, while the CS1 group had significantly more suture failure cases than NS group, these complications were not more common in the CS2 and PS groups than in NS group. These results suggested that more than four weeks of smoking cessation can reduce the rate of suture failure. Our results support the previous findings [17] on inability of short‐term (2–3 weeks) cessation of smoking to reduce the risk of complicated tissue and wound healing in colorectal surgery. High overall postoperative complications in the CS and PS groups are not inconsistent with previous studies, although we did not expect this high number of complications. Therefore, smoking should be prohibited to avoid additional increase in complications.

Duration of hospitalization was significantly longer in CS1 group when compared with NS group, but CS2 and PS groups had intermediate durations between the CS1 and NS, without significant differences from the NS group. These findings indicate that > 4 weeks of smoking cessation before surgery contributes to shorter hospitalization times as opposed less than 4 weeks before surgery. However, some studies have demonstrated the effectiveness of short-term smoking cessation [27, 28]. Previous research showed that respectively ~ 8 and 4 weeks of smoking cessation were required to reduce postoperative respiratory complications after coronary artery bypass surgery [29] and lung resection [30, 31]. In breast reconstruction surgery, preoperative smoking cessation of at least 3 weeks resulted in a similar overall complication rate to nonsmokers [32]. Wound healing following head and neck reconstruction surgery improved with 3 weeks of preoperative smoking cessation [18]. Furthermore, smoking cessation of ≥ 4 weeks reduced complications such as skin flap necrosis [33]. These results demonstrate the effectiveness of short-term smoking cessation. However, in gastrointestinal surgery, as in this study, < 4 weeks of smoking cessation did not reduce complications and hospital stay. Postoperative complications were suggested to decrease with increasing smoking cessation duration [27]. Our study showed that postoperative complications were less likely with longer cessation time among those in the PS group.

Although we did not evaluate mortality rates, previous studies suggested that smoking cessation is advisable to reduce mortality in patients with colon cancer [34–36], and benefits of smoking cessation increase over time [37]. Indeed, we observed that longer smoking cessation duration was associated with fewer complications.

Preoperative smoking cessation could be beneficial, even in gastrointestinal surgery, for which short-term effects of smoking cessation are unclear. Perioperative period is a perfect teachable moment for smoking cessation and provides smokers with the opportunity to cease smoking for long term [38]. In this context, everyone involved in care during the perioperative period is in a position to improve not only short-term surgical outcomes, but also long-term health outcomes and costs. As complications following abdominal surgery are common for both CS and PS groups, it is important to prevent starting smoking in the first place, rather than quitting before surgery.

The study has some limitations. Relatively small number of cases in some groups may have contributed to smaller power to detect some inter-group differences. The preoperative smoking cessation period was confirmed only via interview, while biochemical indicators, such as urinary and salivary cotinine, were not used. Examination of passive smoking and electronic cigarettes was insufficient. We were unable to examine preoperative complications that could affect postoperative complications in detail; however, their effects appeared to be small, because only patients with the American Society of Anesthesia physical status classification of 1/2 were included.

In conclusion, our results showed that although more postoperative complications were observed in CS1, CS2, and PS groups compared to the NS group, only the CS1 group had higher rates of suture failures and longer hospitalization time than NS group. Moreover, the complications decreased significantly with prolonged cessation periods in the PS group. Taken together, our results indicate that some of the complications can be reduced by providing at least 4 weeks of smoking cessation before gastrointestinal surgery.
